# Therapeutische Besonderheiten bei Erkrankungen der Mamillenhaut

**DOI:** 10.1007/s00105-022-05031-3

**Published:** 2022-07-11

**Authors:** Thanh Huong Luu Thi, Adina Eichner, Johannes Wohlrab

**Affiliations:** 1 Universitätsklinik für Dermatologie und Venerologie, Martin-Luther-Universität Halle-Wittenberg, Ernst-Grube-Str. 40, 06097 Halle (Saale), Deutschland; 2 Institut für angewandte Dermatopharmazie, Martin-Luther-Universität Halle-Wittenberg, Halle (Saale), Deutschland

**Keywords:** Mammahaut, Mamille, Brusthaut, Brustwarze, Topische Therapie, Mamma skin, Mamilla, Breast skin, Nipple, Topical treatment

## Abstract

Die Mamillenregion ist durch besondere anatomische Verhältnisse charakterisiert und lässt sich aus dermatologischer Perspektive in Brusthaut, Haut des Brustwarzenhofes (Areola) und Haut der Brustwarze (Papilla mammae) unterteilen. Im klinischen Zusammenhang sind die Brustwarzen häufig während der Stillzeit durch mechanische Beanspruchung, Milieuänderung mit Mazeration durch den Milchfluss sowie durch mikrobielle Erreger alteriert. Zudem besteht hier die Gefahr der Entwicklung einer Mastitis puerperalis. Außerhalb der Schwangerschaft und Stillzeit finden sich gelegentlich an der Mamillenhaut Ekzemerkrankungen, häufig bei atopischer Disposition (atopisches Mamillenekzem) oder als irritatives Kontaktekzem („joggers nipple“). Seltener werden allergische Kontaktekzeme auf Konservierungsstoffe von Topika oder Metallen (Piercings) beobachtet. Auch im Rahmen einer Skabiesinfestation wird eine Beteiligung der Mamillen, insbesondere bei Frauen, regelmäßig beobachtet. Von großer klinischer Bedeutung sind seltene, präinvasive Läsionen eines Mammakarzinoms oder der Morbus Paget der Mamille vom extramammären Typ. Durch die besonderen anatomischen Gegebenheiten ist es naheliegend, dass bei der Anwendung von Topika sich auch spezifische Penetrationsbedingungen ableiten. Experimentelle Untersuchungen an Humanhaut ex vivo legen nahe, dass in Abhängigkeit von der Molmasse und der Löslichkeit des Arzneistoffs sowie des eingesetzten Vehikelsystems eine deutliche Zunahme der kutanen Bioverfügbarkeit, insbesondere an der Brustwarze selbst durch den transpapillären Diffusionsweg, auftreten kann. Dies sollte insbesondere bei der topischen Anwendung von Arzneistoffen mit bekanntem dosisabhängigem Nebenwirkungspotenzial (z. B. Glukokortikoiden) beachtet werden. Allerdings fehlt dafür bisher eine klinische Evidenz.

## Abstract (en)

The nipple region is characterized by special anatomical conditions and from a dermatological perspective can be divided into breast skin, skin of the areola and the skin of the nipple. In a clinical context the nipples are often altered during lactation by mechanical alteration, changes in the environment with maceration by the milk flow and by microbial pathogens. In addition, there is a risk of developing puerperal mastitis. Outside of pregnancy and lactation, eczema diseases are occasionally found on the mammary skin, often with atopic disposition (atopic nipple eczema) or as irritant contact eczema (“joggers nipple”). More rarely, allergic contact eczema is observed from preservatives in topical preparations or metals (piercings). Also, in the context of a scabies infestation involvement of the nipples, especially in women, is regularly observed. Of great clinical importance are rare preinvasive lesions of breast cancer or Paget’s disease of the mamilla of the extramammary type. Due to the special anatomical conditions, it is obvious that specific penetration conditions are also derived from the application of topical substances. Experimental studies on human skin ex vivo suggest that depending on the molecular weight and solubility of the drug as well as the vehicle system used, a significant increase in cutaneous bioavailability, especially on the nipple itself through the transpapillary diffusion pathway, may occur. This should be considered in particular in the topical application of drugs with known potential of dose-dependent side effects (e.g. glucocorticoids); however, there is still no clinical evidence for this.

Die Brust mit der Mamille stellt als Hautanhangsgebilde eine einzigartige Funktionseinheit dar. So besitzt die Brustdrüse geschlechtsübergreifend eine große Bedeutung als sekundäres Geschlechtsmerkmal und erogene Zone. Im soziokulturellen Zusammenhang wird insbesondere die feminine Brust als Symbol der Weiblichkeit und Fruchtbarkeit angesehen. Im laktierenden Zustand kommt dem Sekret der weiblichen Brustdrüse als größter Drüse des Körpers eine besondere Funktion bei der Ernährung des Neugeborenen und Säuglings zu. Dieser besonderen physiologischen Bedeutung der Brustdrüse liegen besondere anatomische Gegebenheiten zugrunde, deren Kenntnisse v. a. dann von Bedeutung sind, wenn es zu pathologischen Veränderungen kommt. In diesem Zusammenhang sind maligne Erkrankungen von besonderer klinischer Relevanz, allem voran die verschiedenen Vorstufen sowie Typen des Mammakarzinoms. Die präinvasiven Läsionen werden entsprechend der Betroffenheit von Zellen der Milchgänge (intraduktale atypische Hyperplasie [ADH], flache epitheliale Atypie [FEA], Duktales carcinoma in situ [DCIS]) oder der Drüsenläppchen (lobuläre intraepitheliale Neoplasie [LIN]) histologisch unterteilt [1]. Diese Vorstufen gehen in unterschiedlicher Häufigkeit in ein invasives Karzinom mit duktalem oder lobulärem Ursprung bzw. weitere seltenere Varianten über.

Aus dermatologischer Perspektive finden sich neben benignen und malignen Tumoren v. a. primär entzündliche oder infektiöse Erkrankungen, die es zu erkennen und zu behandeln gilt. Dabei ergeben sich nicht nur Behandlungsoptionen durch Topika für die epikutane Therapie der äußeren Brustanteile, sondern auch in Form von niedrigviskösen Formulierungen zur antitumoralen oder antiinfektiösen Therapie von Erkrankungen der in der Brustwarze mündenden Milchgänge bzw. des Drüsenkörpers. Um die hierfür bedeutsamen Details therapeutisch gezielt nutzen zu können, ist die Kenntnis der Entwicklungsbiologie, Anatomie und der sich daraus ergebenden Besonderheiten dieser Körperregion Voraussetzung.

## Embryologische und anatomische Grundlagen

In den ersten Wochen der Embryonalentwicklung bilden sich symmetrische, lineare Epithelverdickungen, die sog. „Milchstreifen“, welche als ektodermale Bänder entstehen und von der Achsel bis zur Leiste verlaufen [2, 3]. Die daraus entstehenden Milchleisten bilden sich zurück außer im Pektoralbereich, wo der primäre Milchhügel in der ca. neunten Schwangerschaftswoche bestehen bleibt. Der primäre Milchhügel ist für die Entwicklung der Brustdrüse verantwortlich und bringt sekundäre Knospen hervor, die schließlich das Duktalsystem bilden [4]. Die Entwicklung der Mamille beginnt ca. in der 12. bis 16. Schwangerschaftswoche mit der Differenzierung mesenchymaler Stammzellen in glatte Muskelzellen. Diesem Ereignis folgt die Entwicklung der speziellen apokrinen Drüsen hin zu den Montgomery-Drüsen [5]. In der ersten Phase der Drüsenbildung entwickeln sich zwischen 8 und 12 Milchgänge. Diese Gänge sind in Epidermisnähe mit Talgdrüsen verbunden [3]. Die Differenzierung des Brustparenchyms und die Reifung sowie Pigmentierung der Mamille vollziehen sich um die 32. und dauern bis zur 40. Schwangerschaftswoche an [5].

Die Anatomie der Brust umfasst Haut, Fettgewebe, fibroglanduläres Brustgewebe (Kanäle, Läppchen und unterstützendes fibröses Gewebe) und neurovaskuläre Strukturen, die alle über der Brustwand liegen [3, 6]. In der weiblichen, laktierenden Brust verdoppelt sich, hormonell stimuliert, das aktive Drüsengewebe im Verhältnis zum Fettgewebe. Da der Säugling häufig eine Brust bevorzugt und somit die Milchbildung seitendifferent stimuliert, kann es nach Beendigung der Stillzeit und Rückbildung des Drüsengewebes zu Größenunterschieden im Seitenvergleich kommen [7]. Die Milchsekretion erfolgt durch etwa 7 bis 15 Mündungen der Milchgänge an der Oberfläche der Brustwarze. Unmittelbar unterhalb dieser Öffnungen befinden sich in jedem der Hauptgänge Erweiterungsbereiche, die als Milchsinus bezeichnet werden und während der Laktation eine Speicherfunktion besitzen. Die Hauptausführungsgänge weisen zahlreiche Verzweigungen auf, die jeweils in einem terminalen Ductus lobularis enden, in dem die Milch während der Laktation gebildet wird [2].

Die Brustwarze befindet sich leicht medial und inferior zur Brustmitte über dem Th4-Dermatom. Die Größe und das Aussehen der Brustwarzen (Typologie) sind individuell allerdings sehr variabel [8]. Am häufigsten sind die Brustwarzen leicht schräg zur Achselhöhle hin ausgerichtet, um das Stillen zu erleichtern [2]. Als Warzenhof (Areola) wird der individuell stärker pigmentierte Rundhof, der die Brustwarze umgibt, bezeichnet. Im Allgemeinen ist er eher blasser bei Menschen mit heller Hautfarbe und dunkler bei Menschen mit dunkleren Hauttypen. In der Pubertät und während der Schwangerschaft werden sowohl der Warzenhof als auch die Brustwarze stärker pigmentiert und die Brustwarzen vergrößert. Der Warzenhof kann sich postpartal aufhellen, bleibt aber normalerweise im Vergleich zur Färbung ante graviditatis stärker pigmentiert [9]. Als Sulcus wird eine Falte am Übergang zwischen Warzenhof und aufsteigendem Rand der Brustwarze bezeichnet. Er kann wie eine Falte, ein Grübchen oder eine glatte Kurve der Haut imponieren. Die Oberfläche der Brustwarze weist eine kopfsteinpflasterartige Textur sowie Spalten, die zu den Öffnungen der Ausführungsgänge führen, auf. In diesen Spalten könne sich Schuppen ansammeln, die einen Keratinpfropf ausbilden [10]. Die Hautschicht des Warzenhofes ist in der Regel zwischen 0,5 und 2,0 mm dick. Die Epidermis der Brustwarze geht per continuitatem in das Epithel der Gänge über [3]. Zwischen dem sich subepithelial anschließenden korialen Bindegewebe und dem Brustdrüsengewebe fehlt die Subkutis [11]. Die Mamille enthält außerdem eine Schicht aus umlaufender glatter Muskulatur, apokrinen Schweißdrüsen sowie Haarfollikel, die die Peripherie des Warzenhofes umgeben [12]. Zudem finden sich hier die Montgomery-Drüsen, die ihr Sekret über eigene Ausführungsgänge an der Oberfläche des Warzenhofes über 1–2 mm große Papillen (Morgagni-Tuberkeln) absondern [13]. Sie stellen große Talgdrüsen mit Differenzierungsmerkmalen von Milchdrüsen dar und können ein talgig bis milchiges Sekret bilden, welches als Schmier- und Schutzsekret während der Laktation fungiert [4].

## Häufige Erkrankungen der Mamillen und angrenzender Brustanteile

**Figure 1:**
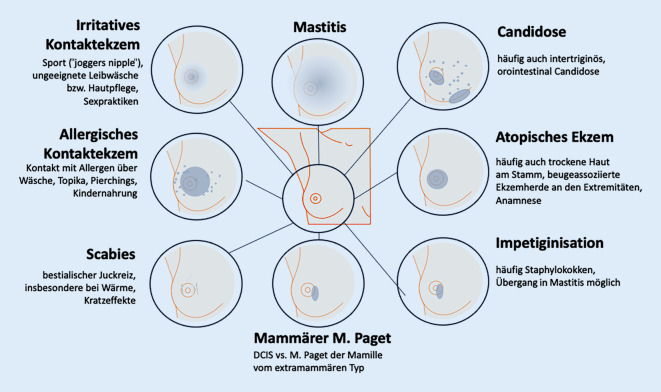

Grundsätzlich können alle dermatologischen Erkrankungen auch die Mamillenregion betreffen. Aufgrund der beschriebenen strukturellen und funktionellen Besonderheiten gibt es aber ein typisches Spektrum von Dermatosen mit besonderer klinischer Relevanz ([4, 14, 15]; Abb. 1).

**Figure 2:**
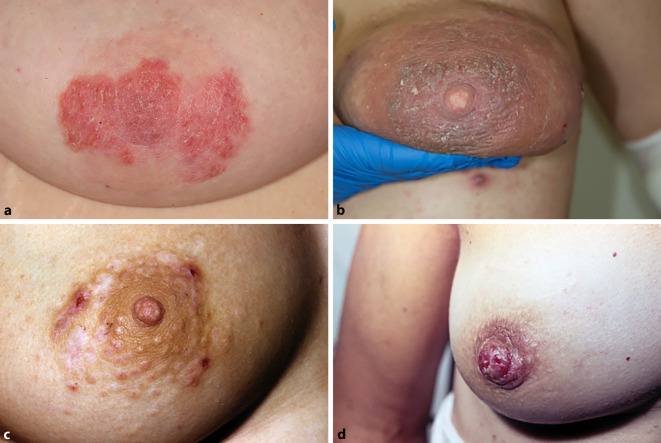

Häufig werden *Ekzemerkrankungen* der Mamillen beobachtet. Besonders prädestiniert hierfür sind junge Frauen mit atopischer Disposition, die während der Pubertät durch Zunahme des Brustvolumens und begleitender Reibung der Mamillen an unzureichend größenangepasster Unterwäsche oder Sportkleidung über Irritation und Juckreiz eine häufig auch staphylogen kolonisierte Ekzemmorphe entwickeln [15]. Vergleichbare Zusammenhänge können auch als atopische Schwangerschaftsdermatose auftreten, wobei hierzu noch die hormonell induzierte Typ-2-gewichtete Immunitätslage in der Schwangerschaft das Auftreten zusätzlich augmentiert [11]. Der klinische Phänotyp kann dabei sehr variabel sein und reicht von umschriebenen Erythemen mit Schuppung oder Lichenifikation bis zu akuter Entzündung mit starker Exoserose und Krustenbildung, schmerzhaften Fissuren oder Erosionen. Meistens sind dabei beide Brustwarzen betroffen. Typische subjektive Symptome sind Brennen und Juckreiz. Im engen pathogenetischen Zusammenhang hierzu kann eine dominant irritative Kontaktdermatitis („joggers nipple“) gesehen werden, die durch intensive mechanische Belastung beim Stillen, durch schlechtsitzende oder materialtechnisch ungeeignete Büstenhalter bzw. durch Friktion der Sportkleidung bei gleichzeitigem Schwitzen verursacht wird ([15]; Abb. 2a). Seltener wird eine allergische Kontaktdermatitis beobachtet, die typischerweise einige Stunden bis zu 3 Tage nach Kontakt mit dem auslösenden Allergen auftritt und Streuphänomene aufweist. Die sonst charakteristischen Exsudativpapeln eines akuten allergischen Kontaktekzems treten an den Mamillen selten auf. Auslösende Allergene sind häufig 5‑Chlor-2-methyl-4-Isothiazolin-3-on, ein gängiger Konservierungsstoff in Waschmitteln und Weichspülern bzw. Nickelsalze bei Brustwarzenpiercings [16]. Seltener werden auch Inhaltsstoffe von Nagellack als Allergene im Epikutantest identifiziert. Bei stillenden Patientinnen kommen als Quellen von Kontaktallergenen zudem Inhaltstoffe von Brustwarzen-Cremes (z. B. Lanolin, Kamille oder Aloe vera) oder auch feste Nahrungsbestandteile in Betracht, die in die Ernährung des Säuglings eingeführt werden [11]. Die Brusthaut und insbesondere die Mamillen sind auch häufiger Infestationsort einer Skabies. Meistens imponiert klinisch ein postskabiöses Ekzem, in dem sich auflichtmikroskopisch gelegentlich auch Milben und deren Grabegänge nachweisen lassen ([17]; Abb. 2c).

Ekzem-lädierte Mamillen bergen zudem die Gefahr einer Kolonisation mit *Staphylococcus aureus* (seltener auch anderen Bakterien) oder bei Stillenden zusätzlich mit *Candida albicans*, die durch Invasion zu einer Infektion der Milchgänge und des Drüsenkörpers führen können ([18]; Abb. 2b). Die sich dadurch entwickelnde Mastitis wird in die häufigere Form der Mastitis puerperalis und der selteneren Mastitis non-puerperalis unterschieden und kann in einen Mamillen- oder Brustabszess übergehen. Die Mastitis puerperalis kann auch aseptisch durch Behinderung des Milchabflusses aus der Brustdrüse bedingt sein und ist die häufigste Ursache zur frühzeitigen Stillbeendigung [19, 20]. Die dabei beobachteten Symptome sind örtlich begrenzte, gerötete, überwärmte und geschwollene Bereiche der Brust sowie lokal starke Schmerzen im Drüsenkörper meist unilateral, in seltenen Fällen aber auch bilateral [21]. Zudem können systemische Symptome wie Unwohlsein und Fieber auftreten. Für Mamillen- oder Brustabszesse ist die Brustwarze die Eintrittspforte der Infektionserreger. Zu den prädisponierenden Faktoren zählen deshalb auch lokale Vorschädigungen, Rhagaden bzw. lokale Traumata bei hohem Brustvolumen [22]. Klinisch zeigt sich eine harte, gelegentlich fluktuierende Masse in der Tiefe des Drüsenkörpers, meist mit ausgedehntem Erythem der darüber liegenden Brusthaut. Schwangerschaft bei Müttern älter als 30 Jahre, Erstschwangerschaft, ein Gestationsalter ≥ 41 Wochen und präexistente Mastitis gelten als Risikofaktoren für die Entwicklung von Brustabszessen in der Stillzeit [20, 21].

Von besonderer klinischer Bedeutung ist der Morbus Paget der Mamille, der erstmals 1874 von Sir James Paget beschrieben wurde und entweder ein duktales *Carcinoma in situ* (DICS) (32,9 %) oder ein invasives Mammakarzinom (53,4 %) darstellt ([23]; Abb. 2d). In selteneren Fällen (13,7 %) wurden auch isolierte Befunde ohne Nachweis von DICS oder Mammakarzinom beschrieben, die als „Morbus Paget der Mamille vom extramammären Typ“ bezeichnet werden. Typischerweise finden sich klinisch unilateral und in Bezug auf die Mamille ein meist asymmetrisches, glänzendes Erythem, welches klinisch nicht sicher von einer Ekzemerkrankung abgegrenzt werden kann. Auch eine symptomatische Besserung durch die topische Applikation eines Glukokortikoids ist kein verlässliches Differenzierungskriterium. Bei Verdacht sollte immer eine Biopsie aus dem läsionalen Bereich erfolgen, um eine maligne Genese sicher auszuschließen. Das histologische Merkmal des Morbus Paget der Mamille ist die nestartige und einzelzellige Infiltration der Epidermis der Mamillenhaut mit glandulär differenzierten Tumorzellen mit feingranulärem Zytoplasma und großen zentral gelegenen Zellkernen mit Kernatypien [4, 24]. Zudem lassen sich durch immunhistochemische Färbungen (z. B. HER2, CK7) relevante Differenzialdiagnosen (Toker-Zell-Hyperplasie, pagetoide Dyskeratose, Klarzellakanthom, nävoide Hyperkeratose) abgrenzen [24]. Seltenere Tumoren der Mamillenregion sind Karzinome, intraduktale Papillome, Mamillenadenome, syringomatöse und mesenchymale Tumoren und das Pseudolymphom der Mamille (Lymphadenosis cutis benigna) [25]. Selten findet sich auch akzessorisches mammäres Gewebe in Form einer Polythelie oder Polymastie [26].

## Besonderheiten der topischen Therapie

Topika werden im Bereich der Mamille als barriereprotektive Basistherapeutika oder im Rahmen der arzneilichen Therapie definierter Mamillenerkrankungen eingesetzt. Basistherapeutische Maßnahmen unter Einbeziehung der Mamillenregion sind fester Bestandteil der evidenzbasierten Therapieempfehlung bei Atopiker:innen [27, 28]. Ziel ist es, die Defizite der verschiedenen Barrierekomponenten der Epidermis zu substituieren und dabei die besonderen regionalen anatomischen und physiologischen Gegebenheiten zu berücksichtigen. Auch wenn es keine Evidenz für spezifische pflegetherapeutische Konzepte für die Mamillenregion bei Atopiker:innen gibt, so ist davon auszugehen, dass wasserhaltige Formulierungen mit saurem pH (4–5,5) sowie einem geeigneten Puffersystem und hygroskopische Substanzen (Humectants) in Kombination mit polaren Lipiden (Phospholipiden oder Ceramiden) für die Anwendung geeignet sind. Wasserfreie Formulierungen sollten insbesondere bei wiederholter Anwendung wegen zu starker Okklusionseffekte gemieden werden [29–31]. Bei Sportler:innen hat sich zur Reduktion mechanischer Irritationen und Scherkraftübertragung von Kleidung auf die Mamille das Abkleben mit einem mäßig haftenden Pflaster bewährt, welches nach der Aktivität wieder entfernt werden kann.

Bezüglich der Mikrobiota der Mamille und möglicher Einflüsse einer Dysbiose auf entzündliche bzw. tumoröse Erkrankungen ist bisher wenig bekannt [32]. Untersuchungen mittels 16s-rRNA-Analysen haben eine stabile Diversität des Mikrobioms der Areolarhaut, aber eine abweichende Diversität im Brustdrüsensekret bei Patientinnen nach Mammakarzinom im Vergleich zu gesunden Probandinnen gezeigt [32]. Insbesondere um bei läsionaler Mamillenhaut einer Kolonisation mit pathogenen Erregern bzw. einer Infektion vorzubeugen, werden lokale Antiseptika eingesetzt. Aus mikrobiologischen und galenischen Gründen haben sich ähnlich wie an der Schleimhaut und Übergangsschleimhaut Polihexanid oder Octenidindihydrochlorid als Antiseptika in halbfesten Formulierungen bewährt [33–35].

Auch bei Schwangeren und Stillenden wird eine Brustpflege zur Säuberung und Konditionierung der Brustwarze für einen optimierten Stillvorgang empfohlen [36–38]. Insbesondere bei irritativen Ekzemen der Mamille oder gar Mastitis puerperalis sind Handlungsempfehlungen für Mütter, Hebammen und betreuende Ärzt:innen in einer Leitlinie formuliert [20]. Neben Empfehlungen zur Mamillenpflege und -reinigung hat die Stilltechnik einen entscheidenden Einfluss auf den Hautzustand der Mamillen. Um diese zu optimieren und eine geeignete Stillposition zu gewährleisten, ist eine Stillberatung der Mütter durch Hebammen sowie Laktationsberaterinnen zu empfehlen [20]. Zudem muss in der Stillphase berücksichtigt werden, dass im Bereich der Mamillenhaut topisch applizierte Arzneistoffe potenziell sowohl über die Muttermilch als auch als Rückstandphase über die Lippen des Säuglings im Kind bioverfügbar werden können.

Wegen des unspezifischen antiproliferativen Potenzials einiger Chemotherapeutika zur Behandlung maligner Tumoren (z. B. Mammakarzinom) treten häufig als unerwünschte Begleiteffekte Hautveränderungen wie Trockenheit, Irritation und Juckreiz auf, die auch die Mamillen einbeziehen können. Um diesen präventiv zu begegnen, wurden Strategien zur protektiven Anwendung von speziellen Pflegetherapeutika entwickelt und klinisch validiert [39]. Auch für die kutanen Reaktionen nach Radiatio bei Mammakarzinom liegen Evidenzen zu pflegetherapeutischen Strategien vor [40].

**Figure 3:**
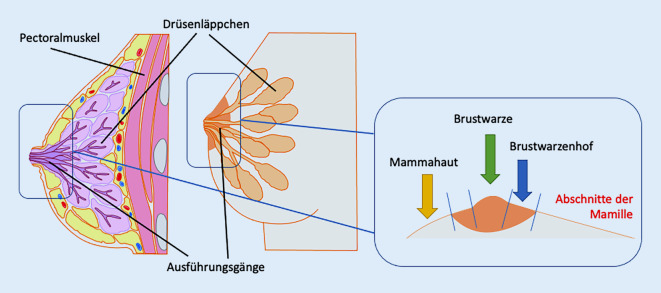

Bei auftretenden entzündlichen, infektiösen oder tumorösen Erkrankungen der Mamille ergibt sich häufig die Indikation einer lokalen Arzneimitteltherapie. Als Arzneistoffe kommen indikationsbezogen vorwiegend Glukokortikoide, Calcineurininhibitoren, Antimykotika, Antiseptika bzw. seltener Antibiotika sowie Immune-Response-Modifier zur Anwendung. Es gibt bisher keine spezifischen Untersuchungen zum Penetrationsverhalten von Arzneistoffen an der Mamille selbst, allerdings lassen die anatomischen Besonderheiten Unterschiede zwischen der Brusthaut, der Haut des Brustwarzenhofes und der Haut der Brustwarze vermuten (Abb. 3). Insbesondere durch den dichten Besatz des Brustwarzenhofes mit Montgomery-Drüsen und den Mündungsöffnungen der Ausführungsgänge der Milchdrüsen an der Brustwarze kann von einer besonderen, zonal unterschiedlichen kutanen Bioverfügbarkeit epikutan applizierter Arzneistoffe ausgegangen werden. Legt man die theoretischen Kenntnisse der Diffusionsrouten durch das Stratum corneum zugrunde, würde man dem Porenweg der Diffusion sowohl im Bereich des Brustwarzenhofes und erst recht an der Brustwarze eine bestimmende Bedeutung beimessen [41, 42]. Allerdings ist unklar, ob diesen regionalen Besonderheiten tatsächlich praktische Relevanz zukommt. Unterscheiden muss man hierbei zwischen den einzelnen Zielkompartimenten. So ist aus dermatologischer Perspektive v. a. die kutane Bioverfügbarkeit zur Behandlung von Dermatosen von Bedeutung. Eine systemische Bioverfügbarkeit hingegen ist wegen der geringen Fläche der Mamillen praktisch zu vernachlässigen. Aus onkologischer Sicht ist darüber hinaus aber auch die duktale Bioverfügbarkeit nach topischer Applikation von Interesse. So ergeben sich durch die Reduktion von unerwünschten Wirkungen im Vergleich zur systemischen Gabe oder durch eine lokal verbesserte Dosis-Wirkungs-Beziehung durchaus Vorteile für die topische Applikation antikarzinomatöser Arzneistoffe [43]. Dabei zielt das galenische Konzept auf eine transpapilläre Arzneistofffreisetzung über die Milchdrüsenausführungsgänge der Brustwarze ab [44, 45]. Anhand von fluoreszenzmarkierten hochmolekularen Modellsubstanzen konnte die verbesserte Bioverfügbarkeit im periduktalen Gewebe über diese Penetrationsroute sowohl im Tierversuch als auch an menschlichem Brustgewebe nachgewiesen werden [46]. Zudem finden sich auch Daten zu Ex-vivo-Untersuchungen der Permeation topisch applizierter hydrophiler und hydrophober Fluoreszenzfarbstoffe in unterschiedlichen Vehikelsystemen zur Charakterisierung dieser besonderen Penetrationsroute [47–50]. Auch wenn die Aussagen aus den bisherigen Untersuchungsdaten nicht einheitlich sind, so kann dennoch festgestellt werden, dass die topische Applikation von antikarzinomatösen Arzneistoffen in frühen Entwicklungsstadien eines duktalen Mammakarzinoms (ADH, FEA, DCIS) bzw. beim Morbus Paget der Mamille vom extramammären Typ eine therapeutische Option darstellen kann. Dies belegen auch tierexperimentelle Daten mit einem als Prodrug veresterten Ciclopiroxolamin-Derivat, bei dem eine antitumoröse Wirkung nachgewiesen wurde [51, 52]. Auch im Bereich der Brusthaut wurde an Ex-vivo-Humanhaut in flächiger Anwendung durch Microneedling und den Einsatz von Mikroemulsionen versucht, Celecoxib topisch zu applizieren, um einen präventiven Effekt bei Mammakarzinomen zu erreichen [53]. Insgesamt fehlt aber für die genannten pharmakokinetischen Strategien bisher die klinische Evidenz.

## Fazit

Insgesamt ist festzuhalten, dass durch den zonalen anatomischen Aufbau der Mamillenregion besondere Penetrationsbedingungen für topisch applizierte Arzneistoffe vorliegen. Aus experimentellen Penetrationsuntersuchungen an Humanhaut mit Modellsubstanzen lassen sich Unterschiede in Abhängigkeit der Molmasse und der Löslichkeit des Arzneistoffes sowie des eingesetzten galenischen Konzeptes bezüglich der kutanen Bioverfügbarkeit insbesondere durch die transpapilläre Penetrationsroute ableiten. Dadurch zeichnet sich ein besonderes Risikopotenzial bei der topischen Behandlung der Brustwarze insbesondere mit Arzneistoffen ab (z. B. Glukokortikoide), die ein bekanntes dosisabhängiges Potenzial für unerwünschte Wirkungen bieten. Mangels klinischer Daten ist bisher allerdings unklar, ob die Charakteristika von Brusthaut, Haut des Brustwarzenhofes und Haut der Brustwarze überhaupt praktisch relevante Unterschiede bedingen. Hierzu sind weiterführende Untersuchungen mit Beispielarzneistoffen notwendig.
